# 30 years of climate related phenological research: themes and trends

**DOI:** 10.1007/s00484-025-02903-w

**Published:** 2025-05-12

**Authors:** Emily J. Hickinbotham, Francesca A. Ridley, Steven P. Rushton, Zarah Pattison

**Affiliations:** 1 Biological and Environmental Sciences, University of Stirling, Stirling, UK; 2 School of Natural and Environmental Sciences, Newcastle University, Newcastle Upon Tyne, England

**Keywords:** Topic modelling, Phenology, Mismatching, Climate change, Transdisciplinary research, Geographic bias

## Abstract

Anthropogenic climate change has caused changes in the seasonal timing (phenology) of life-cycle events with consequential impacts on ecosystem functioning and biodiversity. Over the last 30 years, climate-related phenological research has expanded rapidly. To identify key themes and knowledge gaps in this research landscape we used a text-based analysis approach, topic modelling. Our systematic literature search identified 4,681 publications on phenology between 1989 and 2019. We showed taxonomic and geographic bias in the literature with a large proportion of publications on bird migration and reproduction, insect phenology, marine phenology, and agriculture, focused within the Northern hemisphere. Our results reflected the decadal advances in technology, for example remote sensing studies increased the most in popularity. Topics related to genetics increased along with mismatching, which has impacts on species fitness. While climate-based topics were highly connected, there was little connectivity between different disciplines and newer areas of research. Remote sensing rarely co-occurred with other topics, insect phenology was either being studied with plants or birds instead of being considered as part of a network, and mismatching was rarely studied alongside other methodologies in phenological research. We suggest that transdisciplinary research considering species as part of a system and analyzing new or understudied taxa and regions should be prioritized. The disjuncts identified in this analysis inhibit development of a coherent view of the impact of phenological changes on biodiversity and will have implications for conservation management.

**Supplementary Information:**

The online version contains supplementary material available at 10.1007/s00484-025-02903-w.

## Introduction

Phenology is the study of seasonal timing of life-cycle events for all living organisms on Earth (Rathcke and Lacey 1985). Both biotic and abiotic cues fine-tune phenology to determine an ‘optimal time window’ each year, enabling species to synchronise with important seasonal events (Visser and Gienapp 2019). However, temporal shifts in phenology have occurred as a result of anthropogenic climate change (Memmott et al. 2007; Thackeray et al. 2010; Gronchi et al. 2023; IPCC 2023). There has been an overall advancement of phenological events such as spring phenology, but delays in autumn events have led to an overall lengthening of the growing season (Menzel and Fabian 1999; Abu-Asab et al. 2001; Wolfe et al. 2005; Tansey et al. 2017). Phenological changes have been documented for a selection of taxa in a variety of locations, making them a reliable indicator of the impact of climate change on ecosystems (Walkovszky 1998; Penuelas et al. 2002; Thackeray et al. 2016; Burgess et al. 2018; Bell et al. 2019; Menzel et al. 2020). With changes in climate potentially impacting species fitness as a result of phenological changes, it is timely to synthesise the themes and trends of climate related phenological research to date, to determine future research direction to achieve these targets (Campbell et al. 2009).

The wealth of literature on the impacts of climate change on phenological processes has revealed concerning patterns in species’ phenology. The differences in phenological changes between species and across trophic levels can lead to asynchrony, or ‘mismatching’, where the activities of species become temporally separated (Visser and Gienapp 2019). These differences can be due to the direction and strength of phenological changes. For example, changes in temperature can result in advanced phenology, while changes in precipitation may result in delays, and the strength of these environmental cues can determine whether the overall phenological change is an advance or delay (Ahas et al. 2002; Cleland et al. 2006; Thackeray et al. 2016). Similarly, the magnitude of phenological changes can be different, where the degree of plasticity in species’ phenology determines whether it shifts by days or up to weeks (Memmott et al. 2007). Earlier seasonal phenology has also increased the impact of humans on wildlife. For example, ground-nesting birds in Finland now nest on average 8 days earlier than the sowing date of barley when in the 1970s they would nest several days *after* barley sowing, increasing the risk that their nests will be destroyed by farming equipment (Santangeli et al. 2018).

The variation in phenological response can be influenced by species’ traits; for example, species that respond to environmental cues such as degree days are more likely to experience an advance in their phenology (McMaster and Wilhelm 1997; Hughes 2000; Penuelas et al. 2002; Tansey et al. 2017). Consequently, the inherently higher sensitivity to temperature of ectotherms, or other organisms more reliant on temperature such as poikilothermic insects, mean that the impact of climate change on developmental rates, and thus phenology, of these organisms is expected to be stronger (Hughes 2000; Thackeray et al. 2010; Damien and Tougeron 2019; Bonoan et al. 2021). Ecological generalists utilise a range of environmental cues, making them better equipped to respond and adapt to changes in their environment than species that are specialists and might, for example, depend on the specific timing of a food source (Koerner and Basler 2010; Chmura et al. 2019; Macgregor et al. 2019). Furthermore, the time of year when species are active is important; flowering time is generally found to be more variable in early-flowering plant species, and time of year results in plankton populations showing either large advances or delays in their phenology (Beaubien and Freeland 2000; Hays et al. 2005; Thackeray et al. 2016). Reproductive strategies are also critical: with shorter generation time comes faster microevolution, and short-distance migrants are more responsive to temperatures, while long-distance migrants rely on photoperiod to predict the best time to arrive at breeding grounds (Crick et al. 1997; Bradley et al. 1999; Both and Visser 2001; Thackeray et al. 2010, 2016; Merckx et al. 2021).

The complexity of phenological changes, along with variability in species responses and nonuniform environmental impact from climate change, make this a challenging subject to approach. Indeed, many of the factors expected to influence phenology described in the preceding paragraphs have recorded exceptions (see Chmura et al. 2019). Numerous techniques have been used to monitor and predict the influence of climate change on temporal shifts in phenology (Cleland et al. 2007). The four main avenues of research have either used long-term data (Sparks and Carey 1995; Menzel et al. 2020; Büntgen et al. 2022), remote sensing (Myneni et al. 1997; Schwartz 1998; Beresford et al. 2019; Gu et al. 2023), experimental design such as warming experiments (Arft et al. 1999; Anderson et al. 2023; Chen et al. 2023; de Manincor et al. 2023), and statistical modelling (Chuine et al. 2000; Bascompte and Jordano 2007; Morin et al. 2009; Gronchi et al. 2023). The range in use of methodology has also changed over time due to technological advances. This confers an additional need for better integration of knowledge on phenological changes, but the volume and range of literature makes synthesis difficult, especially when the goal is to get an overview of the key messages (Renner and Zohner 2018).

Topic modelling enables semi-qualitative synthesis of large bodies of literature by using co-occurring patterns of words to determine themes and trends through text analysis of article abstracts (Blei and Lafferty 2009). Due to the semi-automated nature of topic modelling, it enables the common ideas or topics within a body of literature to be summarized rapidly, allowing consolidation of a vast yet diverse research area and offering an alternative to standard literature reviews without the time investment of systematic reviews or meta-analyses (Westgate et al. 2015). Previous research has demonstrated the utility of topic modelling in ecology, with work on conservation science, planning and invasive species (Westgate et al. 2015; Mair et al. 2018; Stevenson et al. 2023).

We used topic modelling to explore broad trends and identify research gaps in the field of phenological responses to climate change over the last 30 years (1989–2019). Our objectives were (1) to identify the most popular areas of phenological research over the last 30 years and assess how topic popularity has changed over time, and (2) assess which areas of phenological research are studied in isolation, and which are commonly linked, to identify key knowledge gaps in this field.

## Methods

We use topic modelling *sensu* Westgate et al. (2015) utilising the code and approach of Mair et al. (2018).

### Literature search and screening

The electronic databases Web of Science and Scopus were searched using the terms: “phenolog*” AND (“global change”| “climate change”| “global warming”) (time-stamped protocol development available at https://osf.io/ghnwp/). Results were included if they were classed as reviews or articles and published in English. This gave an overall result of 12,991 articles (Web of Science = 7296, Scopus = 5695, see Supplementary Fig. 1)).

The results from each database search were imported into EndNote. Duplicate articles were automatically removed using EndNote, after which articles were manually searched for any missed duplicates, leaving 8317 articles. Where possible, abstracts were found for articles with no abstract listed through an internet search of article name and author. Articles for which no abstract could be found were removed. The articles were then screened for inclusion (Supplementary Fig. 1) using the following criteria: articles measured a phenological response or a possible response (whether hypothetical or experimental) to a change in climate or environment (including all variations e.g. wind/ light/ temperature/ moisture/ precipitation/ mineral content/ etc.). The response could be directly measured or could be interpolated/predicted as a response to ongoing anthropogenic climate change. A second reviewer checked 20% of articles randomly sampled using R (R Core Team 2020) to check the abstract inclusion protocol. The agreement between the two reviewers was calculated using Cohen’s kappa.

### Abstract cleaning

Once screened, the articles that met the criteria were imported into R using the *revtools* package (Westgate 2019). Some articles contained both English and French or Spanish abstracts. The non-English abstracts were removed during the screening process in EndNote. Using the package *tm* (Feinerer et al. 2008), abstracts were transformed into a corpus (‘body of literature’) and processed as follows: Search terms, numbers written as words (Grun and Hornik 2011), English stop-words in the *tm* package (Feinerer et al. 2008) and expanded as per Mair et al. (2018), and terms added for copyright reasons by publishers, were removed; Hyphens and forward slashes were changed to spaces and all other punctuation was also removed; The words leftover were reduced to their common root by removing suffixes (also termed stemming), and words that then appeared in five or fewer abstracts were removed completely (Griffiths and Steyvers 2004; Lu et al. 2017).

### Topic modelling

Topics (“a distribution over a fixed vocabulary of terms”, (Blei and Lafferty 2009) were identified using the R package *topicmodels* (Grun and Hornik 2011), by fitting a Latent Dirichlet Allocation (LDA) model with Gibbs sampling (Blei and Jordan 2003). Block-cross validation models were used to indicate the number of topics with the least perplexity, where perplexity is the uncertainty in predicting a single word. Thus, lower perplexity is preferred and the higher the perplexity, the more likely the model is performing no better than chance. The number of topics tested were 10, 20, 30, 40, 50, 60, 100, and 200 (sensu Mair et al. 2018). Perplexity decreased from 40 topics (Supplementary Fig. 2). Therefore, 40 topics represented a sufficient trade-off in perplexity versus representation of the corpus.

The weight, a measure of importance of each word underpinning a topic, declines with an increasing number of words. We used 20 words to describe each topic, as word weight began to decline at approximately 20 words and the top five words represented the highest weight (Supplementary Fig. 3, Supplementary Table 1). These words were used to name topics and classify them into themes (Table 1.) (Westgate et al. 2015; Mair et al. 2018). The five themes identified were: (1) ‘Impact’, topics that are a result of a change in climate or a phenological shift, (2) ‘Plants’, topics which are primarily associated with plants, (3) ‘Climate’, topics that refer to location, weather, and phenological events related to the environment, (4) ‘Scientific approach’, topics associated with the techniques used to assess phenology, and finally (5) ‘Recipients’, topics that refer to a taxonomic group, separate from plants, that are the receivers of an impact.

**Table 1:** The topic number, 5 highest weighted words, topic name and theme

No.	Top 5 words	Topic name	Theme
1	Season, grow, length, end, start	Growing season	Climate
5	Event, increase, frost, extrem, risk	Disruptive events	Climate
6	Precipit, veget, studi, grassland, region	Rain determining SOS	Climate
10	Ecosystem, product, carbon, dynam, atmospher	Ecosystem function	Climate
11	Northern, north, latitude, region, rang	Geographical location	Climate
13	Spring, winter, warm, summer, temperatur	Warming impact on seasonality	Climate
17	Ice, lake, snow, cover, arctic	Ice and snow phenology	Climate
22	Annual, climat, year, variabl, rainfal	Latitude and climate	Climate
30	Water, soil, drought, increase, product	Water stress	Climate
33	Studi, area, differ, urban, result	Urban impacts	Climate
36	Site, elev, high, snowmelt, gradient	Elevation	Climate
39	Temperature, degree, increase, air, mean	Temperature fluctuation	Climate
8	Factor, respons, climat, import, understand	Processes that drive phenology	Impact
14	Ecology, impact, research, system, includ	Ecological impact of changes	Impact
19	Effect, affect, interact, impact, direct	Outcomes	Impact
20	Trend, term, long, show, chang	Trends	Impact
24	Popul, resource, mismatch, declin, size	Consequences of mismatching	Impact
27	Chang, shift, respons, time, advanc	Temporal shifts	Impact
32	Speci, community, respons, among, distribut	Biogeography	Impact
35	Popul, adapt, trait, select, plastic	Environmental genetics	Impact
37	Variat, time, pattern, across, spatial	Spatial-temporal variation	Impact
38	Year, earli, late, period, may	Direction of shift	Impact
40	Day, date, advance, signific, year	Timing	Impact
2	Leaf, Forest, Tree, Decidu, Temper	Plant leaf phenology	Plants
3	Growth, bud, tree, burst, differ	Plant life-history events	Plants
16	Chill, require, temperature, pollen, accumul	Plant chilling and heat requirements	Plants
18	Flower, plant, time, fruit, pollin	Plant-pollinator interactions	Plants
21	Develop, seed, stage, studi, delay	Agricultural phenology	Plants
34	Crop, yield, wheat, period, increas	Agronomy	Plants
7	Migrat, arriv, bird, time, migratori	Bird migration	Recipients
9	Time, sea, bloom, water, phytoplankton	Marine phenology	Recipients
15	Insect, develop, emerg, host, generat	Insect phenology	Recipients
28	Breed, nest, lay, time, egg	Bird reproduction	Recipients
31	Reproduct, time, life, condit, histori	Reproductive plasticity	Recipients
4	Warm, plant, respons, treatment, experi	Plant experiments	Scientific approach
12	Data, observ, use, studi, record	Methodology	Scientific approach
23	Climat, future, scenario, region, project	Modelling future climate change	Scientific approach
25	Use, time, analyzi, analyz, method	Analysis	Scientific approach
26	Model, predict, use, simul, base	Predictive modelling	Scientific approach
29	Veget, green, land, ndvi, use	Remote sensing	Scientific approach

### Topic prevalence over time

The weights for each topic per decade were calculated by summing the weight of the topic for each article each year within each decade (Westgate et al. 2020). The only article from 1989 was removed for this analysis, leaving three decades: 1990s, 2000s, 2010s.

### Topic co-occurrence within articles

The co-occurrence of pairs of topics in abstracts was determined using the distribution of topic weights. The weights for each of the 40 topics within each abstract was log_10_ transformed. Euclidean distances were calculated on a scale from zero, where the pair of topics never co-occurred in the same abstract, to one where there the pair of topics always co-occurred (Westgate et al. 2015).

### Topic generality vs. specificity

The distribution of topic weights within the corpus was used to determine topic generality. General topics represent a concept that applies to a wide range of articles in the corpus, whereas more specific topics apply to only a few. The topic with the highest weight for each article was selected, and the mean weight when selected vs. the mean weight when not selected was plotted (Westgate et al. 2015).

### Geographic bias

The frequency of studies based in each country was determined by searching abstracts for every country name, with additional variations where countries may have multiple names (e.g. “UK”, “Britain”, “USA”, “America”). The number of articles per country was then plotted on a world map.

### Topic popularity

Topic popularity was assessed using both the total number of articles published on each topic, and the change in the number of articles published on each topic over the study period (Westgate et al. 2015). The package *lme4* (Bates et al. 2015) was used to fit a GLMM specified with a Poisson distribution and log-link, with year and topic as explanatory variables and the number of articles per topic per year as the response variable. A popular topic was indicated by a positive random intercept, showing a higher-than-average number of articles published on that topic during the study period. A topic that increased in popularity was indicated by a positive slope showing an increase in articles published on that topic during the study period (Fig. 6) (Westgate et al. 2015).

### Journal publication contribution

Additionally, the publication contribution of journals in the corpus was assessed by quantifying the number of articles per journal. The distribution of articles among topics for the top five journals was then be compared.

## Results

**Fig. 1:**
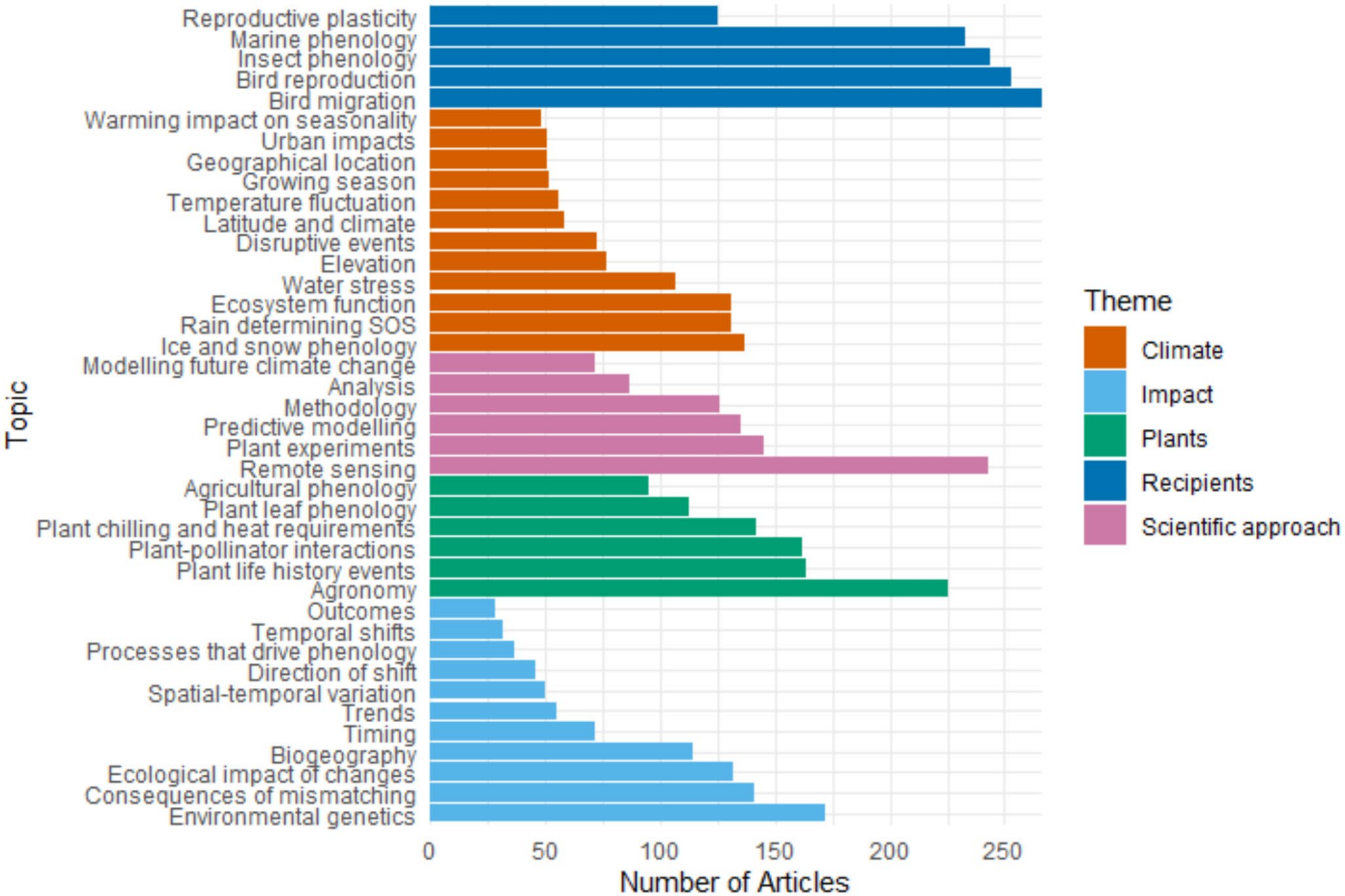
Frequency of topics across the number of articles in the corpus, given the topic weight for each article. Topics are grouped by themes based on their highest weighted words

Of the 8317 articles screened, 4681 articles with 3884 words were included in the analysis. There was moderate agreement between the two reviewers (Cohen’s Kappa = 0.52) (McHugh 2012). The 20 highest weighted words for each of the 40 topics (Supplementary Table 1) were used to name each topic and classify them into one of five overarching themes: ‘Climate’ (12/40), ‘Impact’ (11/40), ‘Plants’ (6/40), ‘Scientific approach’ (6/40), and ‘Recipients’ (5/40) (Table 1). The theme ‘Recipients’ contained topics with some of the highest frequencies of articles (*Bird migration*, *Bird reproduction*, *Insect phenology*, and *Marine phenology*), where these topics had the highest weight in these articles (Fig. 1).

The topics *Remote sensing* in ‘Scientific approach’, *Agronomy* in ‘Plants’, and *Environmental genetics* in ‘Impact’ also had a high frequency of articles. The topics *Analysis* and *Methodology*, and *Agricultural phenology* and *Agronomy*, were conceptually related (*Analysis* contained the word ‘method’) but were still identified as distinct topics by the model. For some topics, the taxa/region commonly used to study them were confounded with the topic subject, such as *Plant leaf phenology* (which included ‘oak’), *Plant life history events* (‘pine’, ‘boreal’), *Rain determining SOS* (‘plateau’, ‘China’), *Geographical location* (‘Europ’, ‘America’), *Plant-pollinator interactions* (‘bee’), *Insect phenology* (‘moth’, ‘butterfli’), *Bird reproduction* (‘tit’), and *Water stress* (‘Mediterranean’) (Supplementary Table 1). (See Supplementary Fig. 4 for similarity between topics and themes).

### Topic prevalence over time

**Fig. 2:**
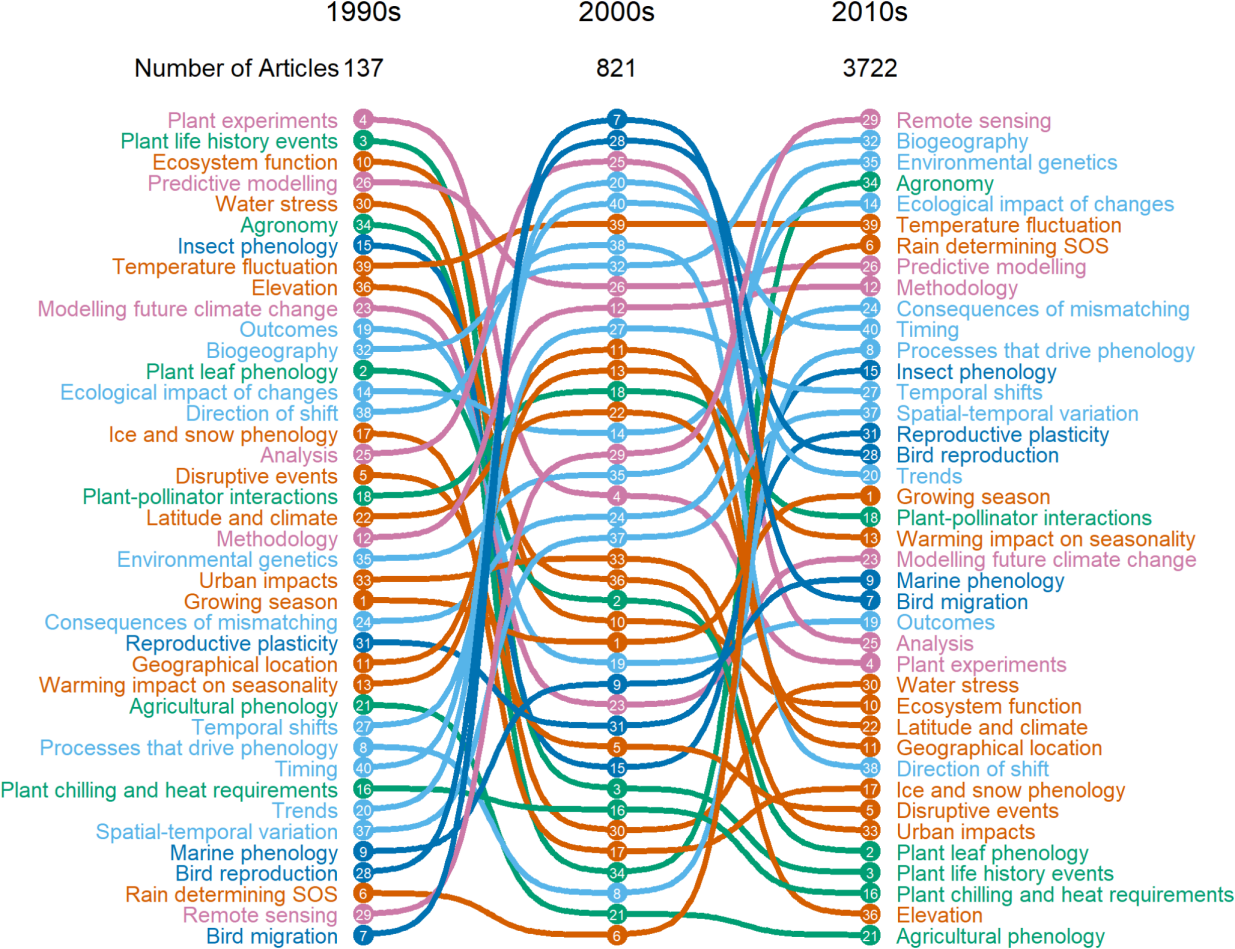
Decadal change in the prevalence of 40 topics. Numbers refer to topic number (Westgate et al. 2020)

The highest weighted topics (Fig. 2) were not consistent across the three decades. The topic *Remote sensing* consistently increased in overall weight, rising from position 39 to 17, and then to position 1 in the 2010s (Fig. 2). Other topics that consistently increased were *Biogeography* (position 12 to 2), *Methodology* (21 to 9), *Environmental genetics* (22 to 3), *Consequences of mismatching* (25 to 10), *Spatial-temporal variation* (35 to 15), and *Marine phenology* (36 to 23) (Fig. 2).

For some topics, changes in popularity was not consistent. The lowest weighted topic in the 1990s, *Bird migration*, switched and became the highest weighted topic in the 2000s, before decreasing again to position 24 in the 2010s (Fig. 2). Similar patterns can be seen for other topics (e.g. *Direction of shift* (15-7-32), *Timing* (32-5-11), *Trends* (34-4-18), and *Bird reproduction* (37-2-17)) (Fig. 2). Conversely, some topics decreased from their 1990 position only to increase in the 2010s (e.g. *Agronomy* (6-37-3), *Insect phenology* (7-32-13), and *Processes that drive phenology* (31-38-12)) (Fig. 2).

Despite an apparent increase in popularity in Fig. 6, the topic *Agricultural phenology* (21) decreased in overall weight, falling from position 29 to 40 across the decades. Other topics that decreased were *Plant life history events* (2 to 37), *Ecosystem function* (3 to 29), *Elevation* (9 to 39), *Plant leaf phenology* (13 to 36), *Disruptive events* (18 to 34), and *Plant chilling and heat requirements* (33 to 38) (Fig. 2).

### Topic co-occurrence within articles

**Fig. 3:**
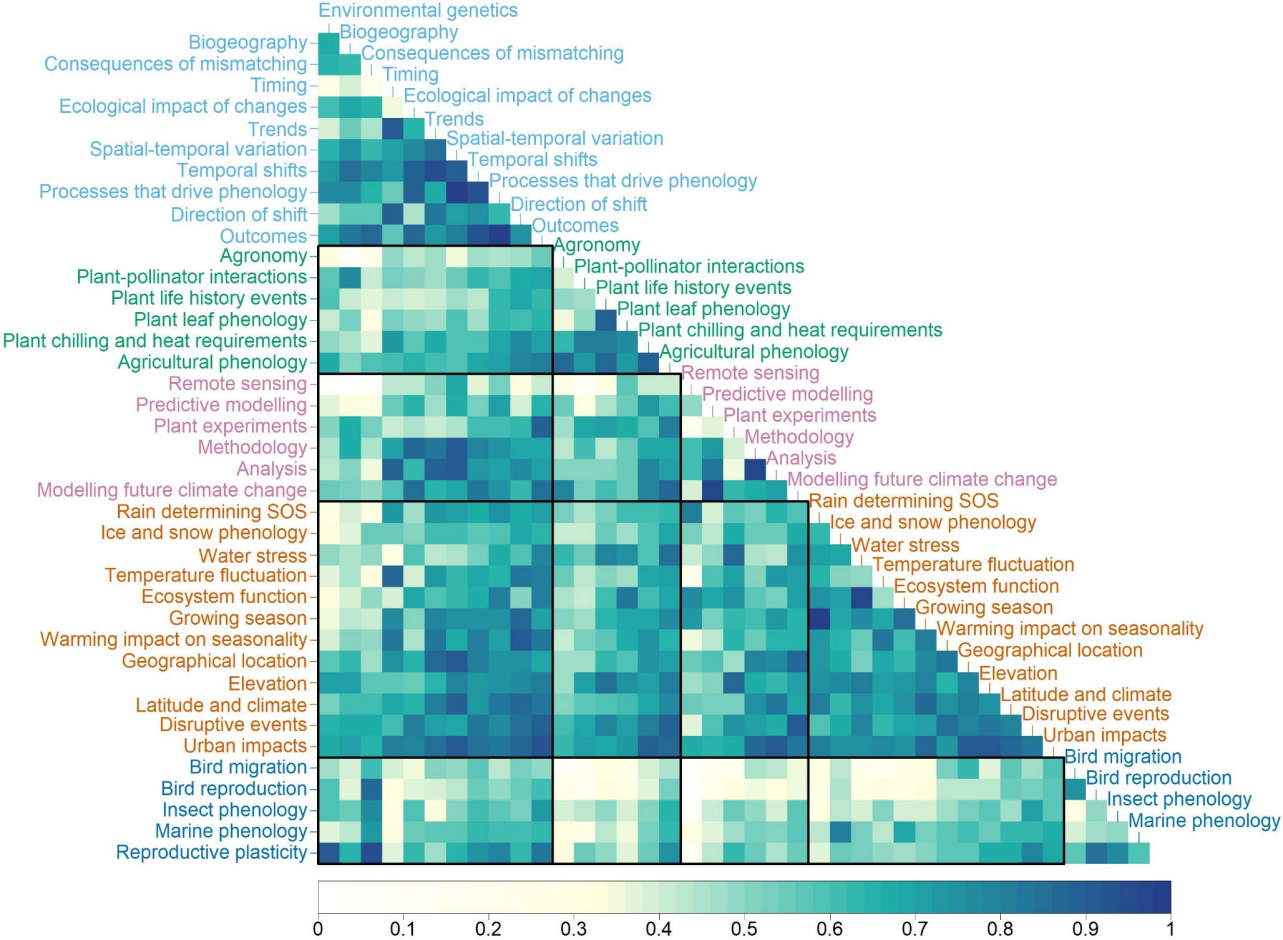
Co-occurrence matrix showing the relationships between topics, sensu (Westgate et al. 2015). Darker blues indicate a stronger relationship, 1 = topics co-occur within articles, 0 = topics do not co-occur within articles. The label colours refer to the topic theme: Light Blue = Impact, Green = Plants, Pink = Scientific Approach, Orange = Climate, Dark Blue = Recipients

Some of the frequently co-occurring topics were due to topic similarity, for example *Predictive modelling* and *Modelling future climate change*, and *Analysis* and *Methodology* (Fig. 3). Within themes, only ‘Climate’ had frequently co-occurring topics, these were: *Rain determining SOS* and *Growing season*, *Elevation* and *Urban impacts*, and *Water stress* and *Ecosystem function* (Fig. 3). Across themes, some important frequently co-occurring topics were *Reproductive plasticity* and *Consequences of mismatching*, and *Disruptive events* and *Modelling future climate change* (Fig. 3). Topics that did not commonly co-occur were also identified in the analysis. The topic *Remote sensing* did not tend to co-occur with most ‘Recipients’ topics, and only rarely co-occurred with ‘Scientific approach’, ‘Impact’, ‘Climate’ and ‘Plants’ topics, with exceptions being *Spatial-temporal variation* and *Rain determining SOS* (Fig. 3).

### Topic generality vs. specificity

**Fig. 4:**
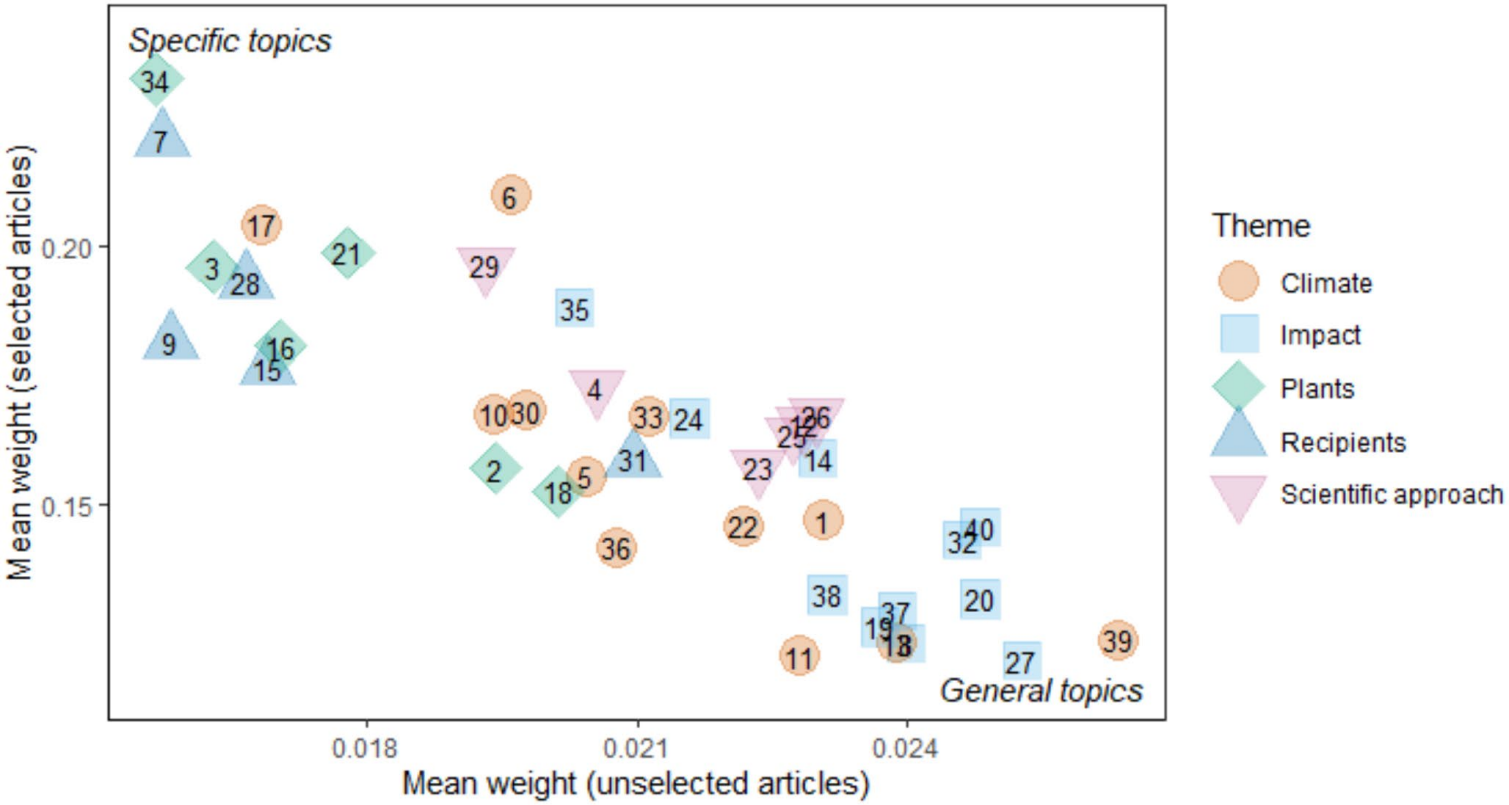
Topic generality, shown by the weight each topic has within articles where it is has the highest weight (selected) vs. when it is not the topic with the highest weight (unselected). Numbers refer to topic number (Table 1.)

Topic specificity is related to topic frequency, as a specific topic is more likely to have the highest weight within any given article. Topics in the themes ‘Climate’ and ‘Scientific approach’ tended to be more general overall, with only the topics *Remote sensing* (29) and *Plant experiments* (4) being more specific (Fig. 4). The most specific topic was *Agronomy* (34), followed by *Bird migration* (7) (Fig. 4). Specific topics were primarily in ‘Recipients’ and ‘Plants’, with only one ‘Climate’ topic, *Ice and snow phenology* (17), being highly specific, indicating this is usually studied in isolation (Fig. 4). Of the ‘Recipients’ topics, only *Reproductive plasticity* (31) was general (Fig. 4). General topics mostly consisted of those in the theme ‘Impact’, but the most general topic *Temperature fluctuation* (39) was in ‘Climate’ (Fig. 4).

### Geographic bias

**Fig. 5:**
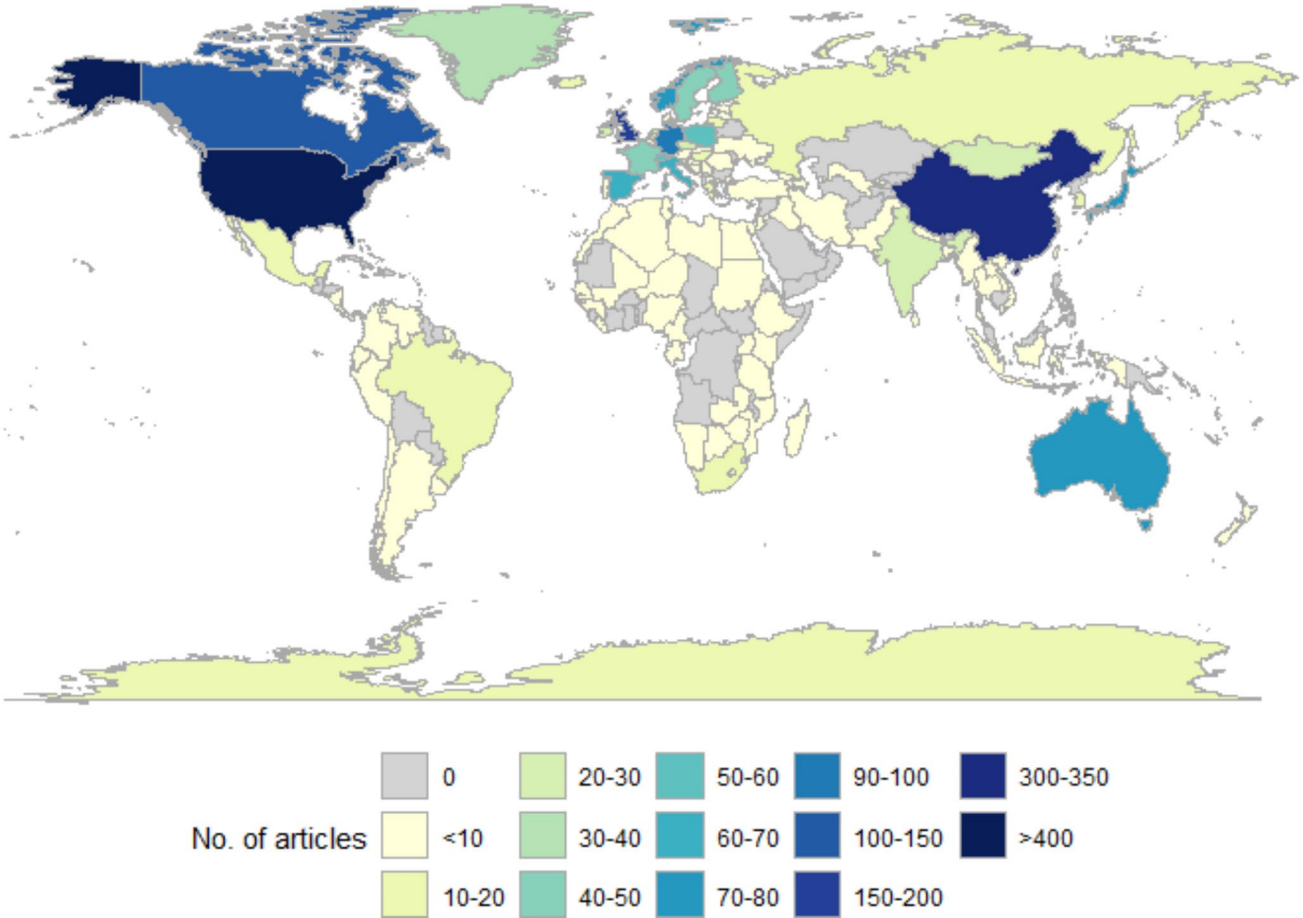
The geographic distribution of published articles included in the study, by frequency per country

The map of article frequency per country revealed large geographic bias in the locations of phenological studies. Generally, there was a bias towards the Northern hemisphere, with a focus on particular countries such as the USA, UK and China (Fig. 5). In the Southern hemisphere, only Australia appears to have a higher frequency of articles, with other countries in the global South commonly having fewer than 20 articles associated with them (Fig. 5). There are also many areas with very little phenological information, particularly towards the tropics (Fig. 5). While it is possible this is due phenological changes not being as clear or consistent as in higher latitudes, and thus not commonly being the subject of phenological research, it is also likely this is also due to research scarcity in these areas.

### Topic popularity

**Fig. 6:**
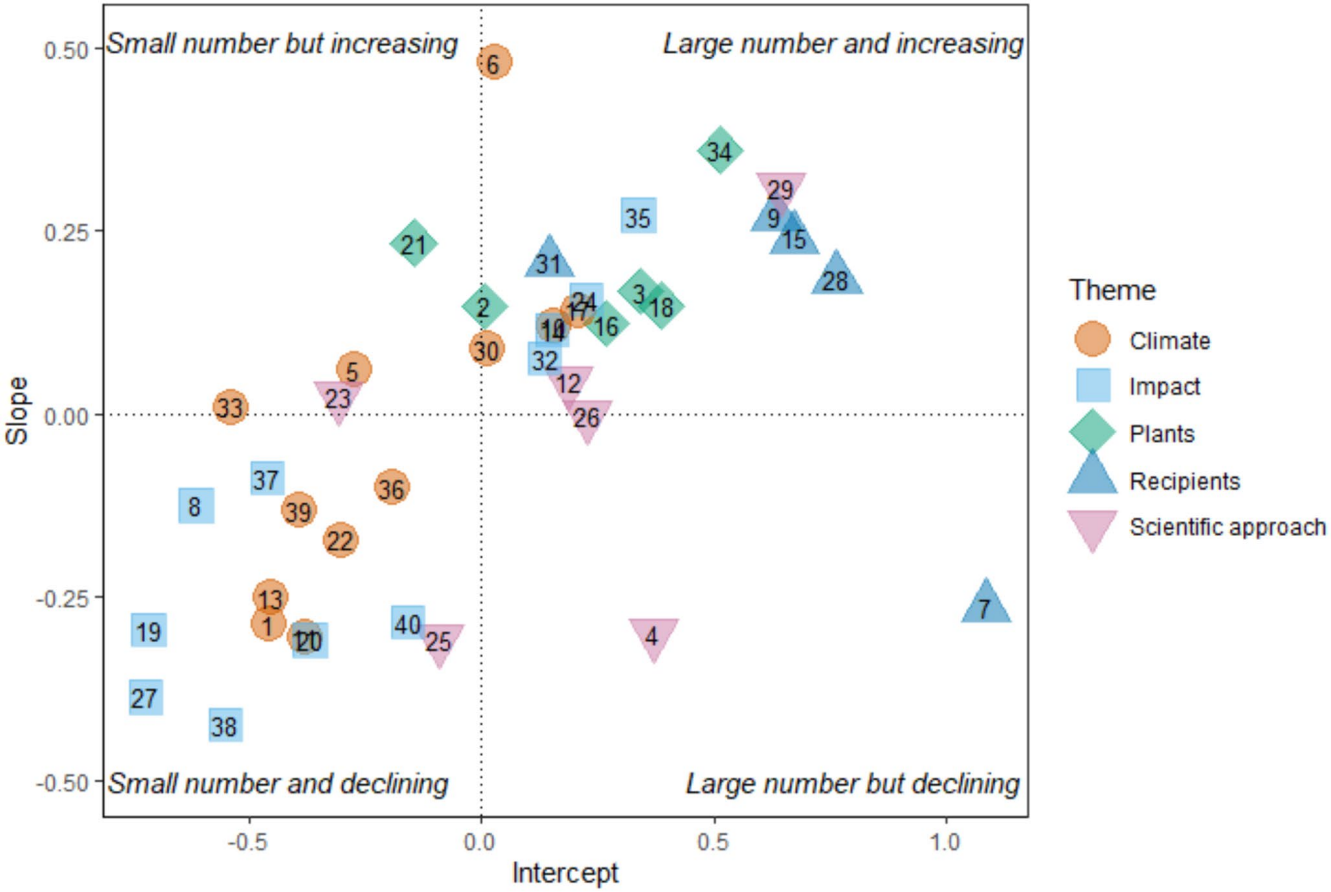
Topic Popularity, shown by the number of articles published across years. Numbers refer to topic number (Table 1.)

Overall, topic popularity is not consistent over time as there was little clustering around a slope of zero (Fig. 6). The topics *Bird migration* (7) and *Plant experiments* (4), which have been frequently published (Fig. 1), were decreasing in popularity (Fig. 6). The topic *Agricultural phenology* (21) appeared to increase in popularity (Fig. 6). There were several “Hot” topics clustered together, where the topic has a high frequency of articles which is increasing, particularly *Rain determining SOS* (6) which had a relatively small intercept but the largest slope, as well as *Agronomy* (34), *Remote sensing* (29), *Environmental genetics* (35), and *Marine phenology* (9) (Fig. 6). The “Cold” topics Direction of shift (38), *Temporal shifts* (27), and *Outcomes* (19), were all in the theme Impacts (Fig. 6).

### Journal publication contribution

**Fig. 7:**
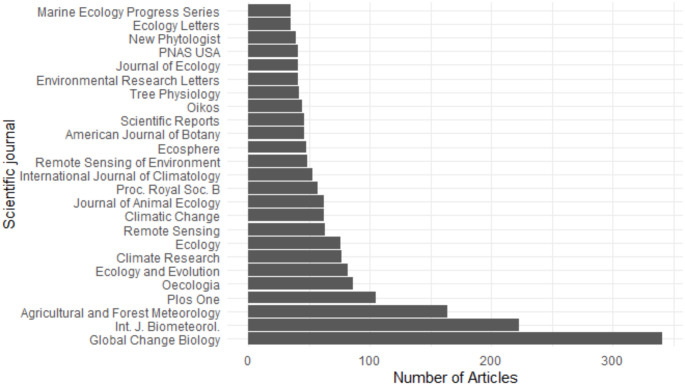
The 25 journals with the highest number of articles

The corpus consisted of 729 journals, and the top 25 contributed 42% of all articles (Fig. 7). Global Change Biology had the largest number of articles (341, Supplementary Fig. 5), primarily publishing on the topics *Marine phenology*, *Plant experiments*, and *Bird migration*, with ‘Recipients’ being the most published theme. The International Journal of Biometeorology (Supplementary Fig. 6) and the journal Agriculture and Forest Meteorology (Supplementary Fig. 7) mostly published within the theme ‘Plants’. The most published topics for the International Journal of Biometeorology were *Plant chilling and heat requirements*, *Plant leaf phenology* and *Analysis*. For the journal Agriculture and Forest Meteorology, only one article was published within the theme ‘Recipients’, and the most published topics were *Agronomy*, *Predictive modelling* and *Plant chilling and heat requirements*. Both PLoS ONE (Supplementary Fig. 8) and Oecologia (Supplementary Fig. 9) published most within the theme ‘Recipients’. The most published topics for PLoS ONE were *Bird reproduction*, *Bird migration* and *Marine phenology*, and for Oecologia were *Insect phenology* and *Bird reproduction* (Supplementary Figs. 8 and 9).

## Discussion

We used topic modelling to quantify the scientific literature on phenology, in the context of climate change, over a 30-year period. Popular topics of research have changed dramatically, with a focus on plant phenology at a regional scale dominating in the 1990s, to the use of novel technologies to assess phenological change at larger spatial scales in the 2010s. Our results show a bias towards study locations, particularly China, Europe, and America (Topics 6 and 11, Supplementary Table 1), as well as study organisms, particularly moths and tit (bird) species (Topics 15 and 28, Supplementary Table 1). There was little inter-connectivity between different methods of studying phenology, or the integration of new ideas, resulting in disconnected research areas. Specifically, remote sensing has become the most popular method of monitoring since the 2010s, however, this has been applied in isolation to previous areas of phenological research (Figs. 2 and 3). Mismatching was also studied in isolation, which can lead to loss of knowledge of the greater impacts of climate change on ecosystems (*Consequences of mismatching*, Fig. 3)

Topic modelling enables rapid synthesis of large diverse bodies of literature using machine learning algorithms. The approach can suffer from “order effects” and is vulnerable to inaccuracies arising from selective reporting and a mismatch between evidence and authors interpretation of evidence, but is useful in revealing broad patterns and changes in literature over time. Limiting articles to specific search terms may have unintentionally excluded research in fields related to phenology that do not explicitly mention “phenology” in their title or abstract. For example, phenological research is inherently linked to climate science, and relevant climatological research – such as that outlined by Schwartz and Crimmins (2025) in their summary of phenoclimatology – could have been overlooked. While this study aimed to assess the interdisciplinarity of phenological research, excluding these related disciplines may have led to the omission of valuable articles. However, broadening the search terms to include all related disciplines would have significantly increased the volume of articles to screen, potentially without improving the accuracy of the results. Further, the agreement between the two reviewers as measured by the Kappa statistic was moderate, likely due to articles that were ambiguous in their use of the word “phenology”. For example, while we were specifically interested in shifts in phenology due to climate change, many articles were measuring the response of plants to climate change where their phenology was listed as a trait. It was for these articles that the two reviewers differed. Their inclusion would not impact the overall results, as they still focused on phenology and climate change.

### Changes in topics over time

As the complex relationship between species’ phenology and environmental heterogeneity is further understood, the focus of research in this field has subsequently changed over the last 30 years (Chmura et al. 2019). In the 1990s popular topics focused on determining the impact of climate change on plants by conducting controlled experiments (position 1, Fig. 2) and studies on phenology predominantly assessing phenological changes in a regional (study area) context with changing climate. Hence topics such as *Latitude and climate*, *Geographical location*, and *Elevation*, which commonly co-occurs with *Urban impacts*, were also popular in the 1990s. Studies utilised the more extreme seasonal temperature changes at higher latitudes or elevations, where there were expected changes in snowmelt date with additional warming.This hypothesises that phenological changes may be more severe for species nearer the poles, yet there has been no consensus on the direction of these shifts (Linhart and Grant 1996; Bradley et al. 1999; Inouye 2008; Chmura et al. 2019).

Following this was an increase in analyses of long-term data, as the possibility of using species’ phenology as a bioindicator of climate change impacts became apparent (De Groot et al. 1995; Sparks and Carey 1995). This is a potential explanation for the rise in popularity of bird focused topics in the 2000s (shown by topics *Bird migration* and *Bird reproduction* in Fig. 2), as both their reproduction and migration are useful phenological indicators, and there are numerous long-term records of these events (De Groot et al. 1995). The variability in insect phenological research and continuing decline of plant research popularity during the 2000s may be a result of this shift in focus to birds phenology with climate change. However, while there is a decline in plant topic popularity, this does not necessarily mean there is an overall decrease in plant research. The number of articles increases substantially in each decade as the study of phenology expands to new species, methodologies and areas of research. Thus, while there is a decrease in plant topics popularity, this is indicative of plant research having a smaller overall topic weight compared to other topics, and not a decrease in plant research overall.

Towards the end of the study period in the 2010s, studies have evolved from focusing on the climate influenced phenological change of one species, to how these changes impact pairwise interactions. (Fig. 2). This ecosystem-view of phenology led to topics related to seasonality (*Warming impact on seasonality*, *Growing season*, and *Rain determining SOS*) increasing in popularity in the 2010s (Fig. 2). Studies have thus evolved from simply quantifying phenological changes in different taxa, to understanding the variation in phenological responses and how this can impact on species interactions, along with their interactions with the landscape (Chmura et al. 2019; Visser and Gienapp 2019). For example, loss of trophic interactions can happen as species track their ideal climate by migrating to higher latitudes or elevations to avoid temporal shifts, thus causing spatial mismatching between trophic levels (Chuine and Beaubien 2001; Penuelas and Boada 2003; Memmott et al. 2007; Thackeray et al. 2010). This is reflected in the shift in geographical topics from the immovable *Latitude and climate* and *Geographical location*, to the more organism-centric *Spatial-temporal variation* and *Biogeography*. Within the same spatial area, species that have ancestral associations, such as a predator-prey or plant-pollinator relationship, can become mismatched temporally due to asynchronous phenology (Memmott et al. 2007; Hegland et al. 2009; see Visser and Gienapp 2019). Furthermore, secondary consumers (predators) seem to have lower climate sensitivity which could exacerbate mismatches as primary consumers and producers benefit from the lost interaction (Thackeray et al. 2010, 2016). This relatively new direction in phenological research led to the topic *Consequences of mismatching* rising 15 places from the 1990s to position 10 in the 2010s (Fig. 2).

The topic *Reproductive plasticity*, which commonly co-occurs with *Consequences of mismatching*, also increased in popularity in the 2010s (Fig. 2). While mismatching can be advantageous through lessening or removing an antagonistic interaction with a predator, it is possible that natural selection could spur resynchronisation for species in mutualistic interactions due to impacts of mismatching on fitness (Vanschaik et al. 1993; Renner and Zohner 2018; Damien and Tougeron 2019; Visser and Gienapp 2019). However, phenotypic selection on phenology may not be strong enough for resynchronisation to occur, especially for generalist species or those with long generation times (Kingsolver et al. 2001; Memmott et al. 2007). A resultant local genetic differentiation could mean loss of intra-species synchronisation across populations (Linhart and Grant 1996). Thus, this increased focus on species-species interactions and the potential impacts of mismatching on fitness spurred the involvement of genetics, and the topic *Environmental genetics* increased in popularity from position 22 to 3 from the 1990s to the 2010s (Fig. 2).

### Popularity of research topics

Remote sensing increased in popularity, from position 39 in the 1990s to one in the 2010s. The popularity of remote sensing studies is likely in part due to an increase in all publishing on climate change related phenology, as well as the introduction of new technology, particularly the Moderate Resolution Imaging Spectroradiometer (MODIS), which made phenological remote sensing studies more accurate and easier to conduct (Kang et al. 2003). Four methodology types were highlighted topic model in the analysis: *Plant experiments*, *Modelling future climate change*, *Predictive modelling*, and *Remote sensing*. While *Plant experiments* and *Modelling future climate change* did co-occur with some frequency, likely because plant experiments were simulating climate change, there was little overlap with the other two methods (Fig. 3). This is partly to be expected, due to the restrictions imposed by the methodology of remote sensing (Kang et al. 2003).

Within the top 5 journals there was a split in focus, where some journals primarily published plant-related topics (Supplementary Figs. 5 and 6), while others focused on fauna (Supplementary Figs. 4,7 and 8). However, a drawback of this analysis is that topic popularity is intrinsically linked with topic specificity. As the organism being studied is more likely to become the highest-weighted topic in an article, the topics with the highest number of articles were all related to organisms (Bird migration, Bird reproduction, Insect phenology, Marine phenology, and Agronomy), except for remote sensing, which is also specific (Fig. 1). While Fig. 6. Shows whether the number of articles with a topic as the highest weight has increased over time, it does not show this in proportion to the total number of articles published. For example, the increase in *Agricultural phenology* in Fig. 6 is likely due to an overall increase in the number of articles published on the subject (Fig. 2). However, the decline of *Agricultural phenology* in Fig. 2 indicates that despite this increase in *Agricultural phenology* popularity, it was still a small proportion of all research into phenology. This emphasises that limiting an article to one topic could be misleading. However, Stevenson et al. Stevenson et al. (2023) demonstrated that increasing the number of topics assigned using a cumulative threshold approach does not alter the overall conclusions and can obscure overall trends. Additionally, the technique used to identify topics in topic modelling is semi-qualitative. While the analysis brings out the top 20 words associated with the prescribed number of topics, the topic names are decided by the researchers, which introduces bias and a descriptive quality to the model. Alongside this is positive publication bias, where studies that show phenological changes due to climate change are more likely to be published, which influences any summary we may make on the subject (Hughes 2000; Thackeray et al. 2010).

### Research bias

There is a lack of connectivity between some topics, particularly reflected by research on insect phenology. The topic *Insect phenology* commonly co-occurs with mismatching and bird topics, yet it rarely co-occurs with plant-based topics (Fig. 3). This is surprising, considering the model identified a topic on the relationship between insects and plants, *Plant-pollinator interactions* which does commonly co-occur with plant-based topics, particularly agriculture. Evidently, there is a disparity in the way insect phenology is studied within phenological research in the context of climate change; it is either considered as the plant-pollinator or the predator-prey relationship, rarely both. This results in insect and plant phenology being studied multiple times in different contexts with little crossover, which could lead to fragmentation of knowledge across different disciplines for similar study organisms. Similarly, the topics *Bird migration* and *Bird reproduction* did not frequently co-occur with *Plant-pollinator interactions* despite evidence of multi-trophic consequences of shifts in phenology (Burgess et al. 2018). Despite the transition to the population- and ecosystem-view of phenology, impacts are commonly considered in a pairwise manner. Loss of synchrony will undoubtedly have impacts on an entire ecosystem and consequently biodiversity, thus, species need to be considered together as part of a network, such as the oak-caterpillar-bird and sycamore-aphid-parasitoid systems in the UK (Burgess et al. 2018; Senior et al. 2020). In these mutualistic communities phenological timing can be crucial for the management and conservation of these ecosystem interactions (Morellato et al. 2016).

Further, new areas of research are not well integrated with the rest of phenological research, illustrated in Fig. 3. The topic *Consequences of mismatching* rarely co-occurs with most plant topics, the exception being *Agricultural phenology*. This indicates that while mismatching studies have increased in popularity (Fig. 6), they rarely consider the impacts of mismatching with the lowest trophic level, the primary producers. This is concerning, considering that primary producers tend to have stronger alterations to their phenology in response to environmental changes, likely in part due to their inability to move among microhabitats to track their ideal climate (Thackeray et al. 2010). The only ‘Climate’ topics *Consequences of mismatching* tend to co-occur with are those that involve changes in temperature due to geography, and it strongly co-occurs with the ‘Recipients’ topics. This indicates that mismatching studies have focused on fauna and food production, and how geographical changes can impact their phenology and cause mismatching. However, the additional lack of co-occurrence with other methodologies in phenological research means that we are not fully utilising the tools at our disposal to detect this phenomenon. This supports the existing calls (see Renner and Zohner 2018) for transdisciplinary work and integration of data.

In each article, the organism being studied is more likely to become the highest-weighted topic, due to high word frequency specific to that organism. Thus, the high frequency of the ‘Recipients’ topics (Fig. 1) and their top 20 words (Supplementary Table 1), as well as the necessity for an entire theme dedicated to plants, indicate that the common study organisms for phenological studies are: agricultural crops, birds (particularly migratory), insects whose development is clearly impacted by temperature such as butterflies and moths, and phytoplankton in marine studies. This is exemplified by the topic *Bird reproduction*, which contained the word “tit”, a common group of study organisms (Supplementary Table 1). However, there are problems with basing predictions of possible ecological impacts on the phenological changes of a few species. Underrepresentation of other taxonomic groups will hinder our ability to predict how phenological changes will impact ecosystems with further climate change.

In terms of geographical data disparities, there is also a bias towards study areas in the Northern hemisphere, as within the 20 highest-weighted words for the topics *Geographical locations* and *Rain determining SOS* were Europe, America, China, and “plateau”, which refers to the Tibetan Plateau (Supplementary Table 1). This is also evidenced by the number of articles per study location (Fig. 5), with all countries containing more than 100 articles in the Northern hemisphere. In the global South, Australia is the only country that has more than 70 articles (Fig. 5). Increasing evidence of mismatching destabilizing trophic interactions means these biases need to be addressed; despite the likelihood that phenological changes will occur more often in Northern latitudes due to stronger warming, phenological changes are still expected to occur nearer the tropics (Parmesan 2007). A lack of studies from the global South mean we are less likely to understand how phenological changes may impact ecosystems, as studies based on species in the Northern hemisphere may not be applicable to species in the Southern hemisphere. Study bias towards locations and species, and especially the lack of studies near the tropics (Fig. 5), prevents us from being able to predict how trophic interactions may be impacted by phenological changes for vulnerable species as climate changes become more severe.

## Conclusions

Over a 30-year period, the scientific literature on climate related phenological changes has expanded substantially. However, the connectivity between different spheres of research has not followed suit. Different methodologies and research foci have led to a fragmentation of phenological research, where the same topic is studied multiple times in isolation. There are common study organisms and areas that dominate the literature: Migratory birds, crops, insects, and phytoplankton are frequently the focus. While studies that investigate pairwise interactions are useful, these interactions are unlikely to represent an ecological network, and the impacts that phenological changes may have on them. The majority of phenological studies are conducted in the Northern hemisphere. To get a more comprehensive view of the global impacts of phenological changes, we need to first focus on research in the global South. Transdisciplinary research needs to become the norm, both to aid progress towards CBD targets and to improve our understanding of the ramifications of these responses to global climate change.

## Electronic supplementary material

Below is the link to the electronic supplementary material.

Supplementary Material 1

## Data Availability

The a priori protocol, data and code are available in the Topic Modelling repository, https://osf.io/ghnwp/.
