# Supplementary material for: 30 years of climate related phenological research: themes and trends
Source: Int J Biometeorol. 2025 May 12;69(6):1459–73. doi: 10.1007/s00484-025-02903-w (PMC12141420; doi:10.1007/s00484-025-02903-w)
Supplement: Supplementary file 1 — Supplementary Material 1 [file 484_2025_2903_MOESM1_ESM.docx]

Title: 30 years of climate related phenological research: themes and trends

International Journal of Biometeorology

Emily J. Hickinbotham^1^*, Francesca A. Ridley^2^, Steven P. Rushton^2^, Zarah Pattison^1^

Affiliations: ^1^ Biological and Environmental Sciences, University of Stirling, Stirling, ^2^ School of Natural and Environmental Sciences, Newcastle University, Newcastle Upon Tyne.

*Corresponding author: [emily.hickinbotham@stir.ac.uk](mailto:emily.hickinbotham@stir.ac.uk)

# Supplementary Information


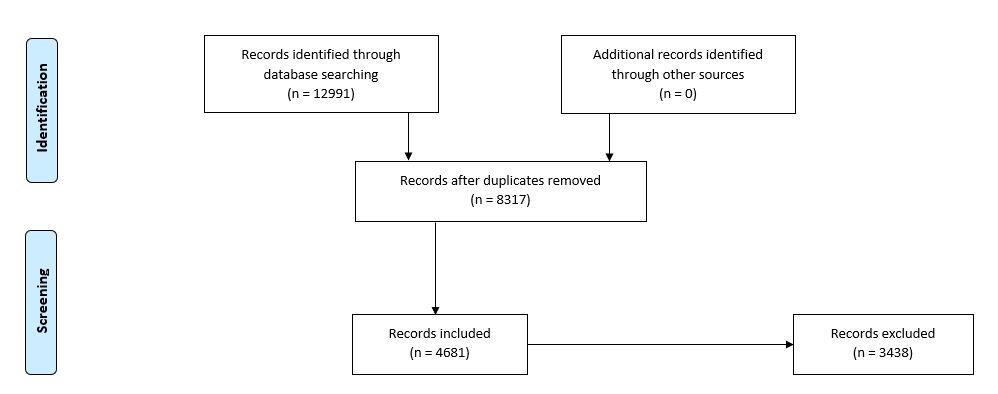


**Figure** **1:** A modified PRISMA diagram of the sorting process of studies for the topic model. Every study was sorted into include/exclude based on the following criteria: articles measured a phenological response or a possible response (whether hypothetical or experimental) to a change in climate or environment (including all variations e.g. wind/ light/ temperature/ moisture/ precipitation/ mineral content/ etc.). The response could be directly measured or could be interpolated/predicted as a response to ongoing anthropogenic climate change..

**Table** **1:** The 20 highest weighted words for the 40 topics.

| No. | Topic 1 | Topic 2 | Topic 3 | Topic 4 | Topic 5 | Topic 6 | Topic 7 | Topic 8 | Topic 9 | Topic 10 |
| --- | --- | --- | --- | --- | --- | --- | --- | --- | --- | --- |
| 1 | season | leaf | growth | warm | event | precipit | migrat | factor | time | ecosystem |
| 2 | grow | forest | bud | plant | increas | veget | arriv | respons | sea | product |
| 3 | length | tree | tree | respons | frost | studi | bird | climat | bloom | carbon |
| 4 | end | decidu | burst | treatment | extrem | grassland | time | import | water | dynam |
| 5 | start | temper | differ | experi | risk | region | migratori | understand | phytoplankton | atmospher |
| 6 | period | senesc | condit | experiment | damag | signific | migrant | environment | peak | cycl |
| 7 | longer | observ | proven | increas | frequenc | delay | distanc | role | abund | result |
| 8 | begin | fall | seedl | growth | sever | correl | speci | influenc | spawn | primari |
| 9 | day | leav | break | effect | weather | zone | breed | photoperiod | fish | terrestri |
| 10 | extend | canopi | spruce | control | reduc | sos | ground | driver | marin | global |
| 11 | earlier | unfold | shoot | shrub | occur | plateau | spring | mechan | zooplankton | activ |
| 12 | durat | color | format | plot | may | differ | winter | process | ocean | increas |
| 13 | increas | autumn | rate | field | decreas | area | date | key | coastal | net |
| 14 | onset | oak | wood | ambient | due | show | earlier | limit | atlant | function |
| 15 | lengthen | studi | stem | manipul | occurr | china | long | studi | concentr | annual |
| 16 | result | time | pine | open | caus | advanc | nao | well | biomass | process |
| 17 | later | evergreen | height | signific | potenti | trend | advanc | determin | earlier | system |
| 18 | extens | result | cessat | year | like | chang | departur | critic | chang | flux |
| 19 | shorter | found | xylem | biomass | result | climat | short | suggest | nutrient | season |
| 20 | prolong | show | boreal | suggest | lead | alpin | condit | play | river | level |

The 20 highest weighted words for the 40 topics.

| No. | Topic 11 | Topic 12 | Topic 13 | Topic 14 | Topic 15 | Topic 16 | Topic 17 | Topic 18 | Topic 19 | Topic 20 |
| --- | --- | --- | --- | --- | --- | --- | --- | --- | --- | --- |
| 1 | northern | data | spring | ecolog | insect | chill | ice | flower | effect | trend |
| 2 | north | observ | winter | impact | develop | requir | lake | plant | affect | term |
| 3 | latitud | use | warm | research | emerg | temperatur | snow | time | interact | long |
| 4 | region | studi | summer | system | host | pollen | cover | fruit | impact | show |
| 5 | rang | record | temperatur | includ | generat | accumul | arctic | pollin | direct | chang |
| 6 | southern | provid | onset | biolog | temperatur | dormanc | durat | reproduct | import | decad |
| 7 | south | collect | autumn | chang | pest | forc | chang | seed | negat | period |
| 8 | europ | monitor | earlier | manag | adult | budburst | freez | result | may | signific |
| 9 | distribut | detect | warmer | particular | butterfli | heat | melt | may | combin | year |
| 10 | central | research | delay | need | may | differ | depth | earlier | howev | observ |
| 11 | eastern | inform | advanc | review | flight | birch | air | set | relat | increas |
| 12 | across | network | climat | current | rate | bloom | result | bee | depend | recent |
| 13 | western | measur | cold | ecosystem | larval | period | increas | speci | addit | studi |
| 14 | similar | indic | suggest | discuss | thermal | phase | region | floral | posit | analyz |
| 15 | geograph | avail | previous | global | development | tree | decreas | durat | climat | last |
| 16 | america | sampl | may | divers | larva | date | free | affect | studi | toward |
| 17 | hemispher | present | result | mani | affect | determin | use | alter | non | past |
| 18 | state | first | less | import | diapaus | studi | surfac | success | consid | earlier |
| 19 | locat | histor | later | focus | stage | releas | break | visit | differ | howev |
| 20 | part | assess | contrast | major | moth | cherri | strong | fungal | indirect | record |

The 20 highest weighted words for the 40 topics.

| No. | Topic 21 | Topic 22 | Topic 23 | Topic 24 | Topic 25 | Topic 26 | Topic 27 | Topic 28 | Topic 29 | Topic 30 |
| --- | --- | --- | --- | --- | --- | --- | --- | --- | --- | --- |
| 1 | develop | annual | climat | popul | use | model | chang | breed | veget | water |
| 2 | seed | climat | futur | resourc | time | predict | shift | nest | green | soil |
| 3 | stage | year | scenario | mismatch | analyzi | use | respons | lay | land | drought |
| 4 | studi | variabl | region | declin | analyz | simul | time | time | ndvi | increas |
| 5 | delay | rainfal | project | size | method | base | advanc | egg | use | product |
| 6 | harvest | period | use | abund | seri | data | consequ | food | index | growth |
| 7 | germin | weather | impact | level | data | develop | recent | date | satellit | condit |
| 8 | compar | tropic | condit | habitat | correl | observ | caus | success | deriv | stress |
| 9 | condit | variat | current | densiti | linear | paramet | report | hatch | base | root |
| 10 | matur | influenc | model | rate | regress | estim | suggest | bird | resolut | reduc |
| 11 | result | relat | assess | predat | relationship | accur | evid | clutch | surfac | dri |
| 12 | qualiti | correl | expect | trophic | statist | improv | studi | peak | data | avail |
| 13 | aim | month | potenti | bodi | indic | valid | find | reproduct | remot | decreas |
| 14 | produc | pattern | centuri | may | variabl | evalu | mani | size | sens | moistur |
| 15 | time | inter | rcp | avail | phase | perform | whether | tit | sos | plant |
| 16 | signific | studi | general | prey | estim | approach | document | relat | differ | biomass |
| 17 | varieti | posit | simul | food | mean | daili | investig | avail | modi | mediterranean |
| 18 | number | indic | suitabl | high | result | appli | like | earlier | imag | nitrogen |
| 19 | evalu | cycl | indic | dynam | show | process | examin | brood | spatial | limit |
| 20 | Higher | fluctuat | repres | forag | approach | calibr | although | may | normal | precipit |

The 20 highest weighted words for the 40 topics.

| No. | Topic 31 | Topic 32 | Topic 33 | Topic 34 | Topic 35 | Topic 36 | Topic 37 | Topic 38 | Topic 39 | Topic 40 |
| --- | --- | --- | --- | --- | --- | --- | --- | --- | --- | --- |
| 1 | reproduct | speci | studi | crop | popul | site | variat | year | temperatur | day |
| 2 | time | communiti | area | yield | adapt | elev | time | earli | degre | date |
| 3 | life | respons | differ | wheat | trait | high | pattern | late | increas | advanc |
| 4 | condit | among | urban | period | select | snowmelt | across | period | air | signific |
| 5 | histori | distribut | result | increas | plastic | gradient | spatial | may | mean | year |
| 6 | environment | shift | signific | cultivar | genet | low | scale | month | sensit | mean |
| 7 | individu | group | show | growth | respons | higher | tempor | april | thermal | averag |
| 8 | femal | rang | found | impact | variat | along | variabl | occur | day | per |
| 9 | behavior | nativ | compar | product | phenotyp | lower | larg | mid | maximum | first |
| 10 | environ | relat | type | rice | environment | differ | local | appear | rise | phenophas |
| 11 | organ | composit | influenc | matur | fit | altitud | climat | first | daili | decad |
| 12 | male | specif | use | agricultur | differ | mountain | within | march | minimum | period |
| 13 | cycl | invas | intens | maiz | local | alpin | vari | number | higher | respect |
| 14 | may | respond | investig | use | evolutionari | limit | respons | time | rate | observ |
| 15 | influenc | across | import | sow | individu | less | relationship | averag | averag | earlier |
| 16 | predict | mani | island | adapt | potenti | relat | among | mean | respons | station |
| 17 | activ | predict | small | decreas | environ | howev | explain | later | night | correl |
| 18 | strategi | common | previous | stage | among | occur | differ | earlier | cool | delay |
| 19 | sex | greater | citi | grain | genotyp | altitudin | geograph | week | warmer | gdd |
| 20 | anim | ecolog | local | durat | natur | found | consist | june | effect | show |


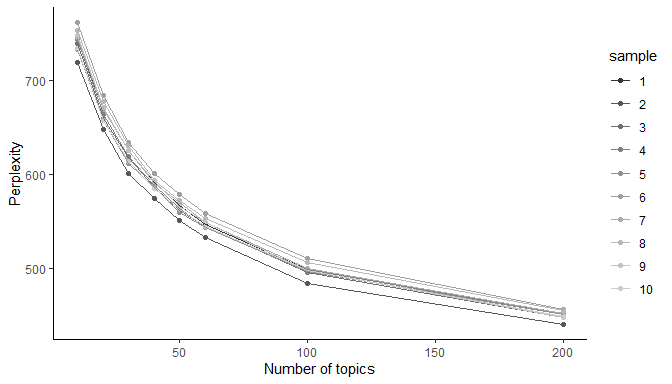


**Figure** **2:** The perplexity (uncertainty in predicting a single word) against the number of topics for a 10-fold block cross-validation.


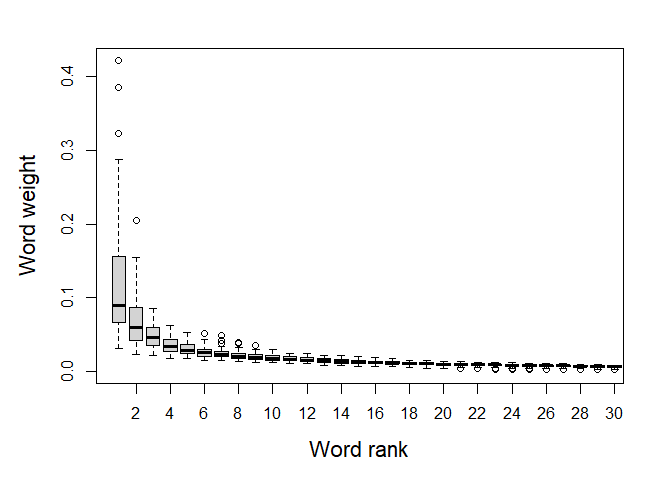


**Figure** **3:** The weight of each word against word rank for the 30 highest weighted words within each of the 40 topics.


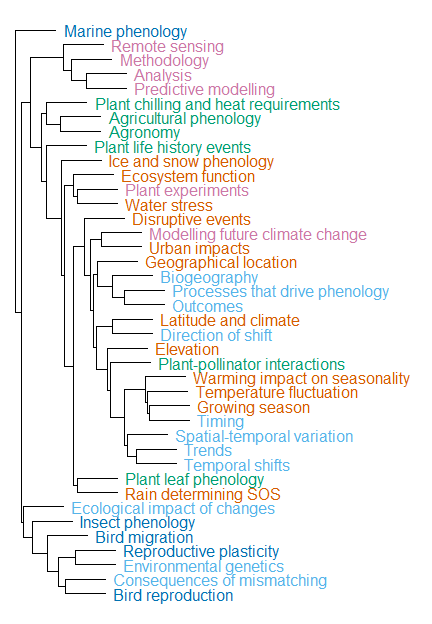


**Figure** **4:** Dendrogram showing topic similarity based on shared occurrence of words.

## Journal plots


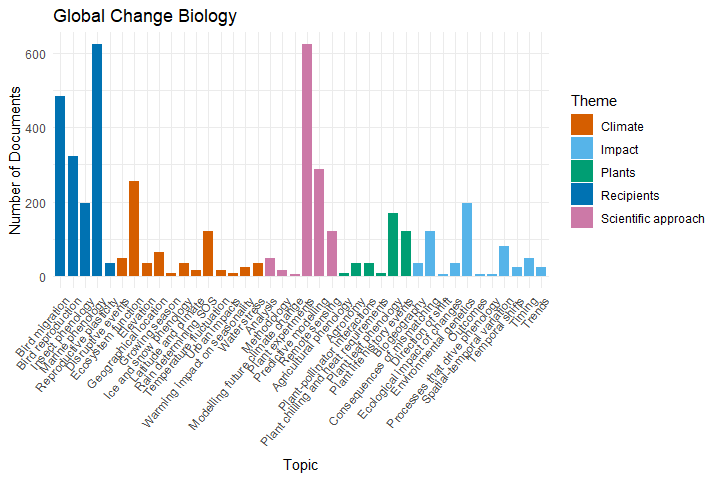


**Figure** **5:** The distribution of topics and themes among 341 documents for the journal Global Change Biology.


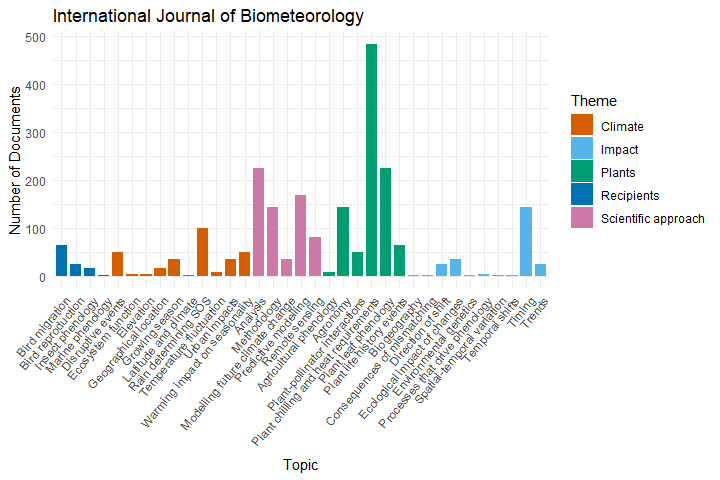


**Figure** **6:** The distribution of topics and themes among 223 documents for the International Journal of Biometeorology.


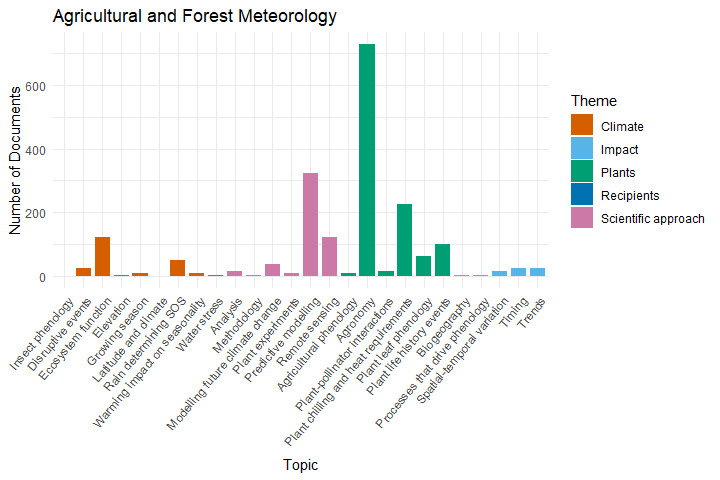


**Figure** **7:** The distribution of topics and themes among 164 documents for the journal Agricultural and Forest Meteorology.


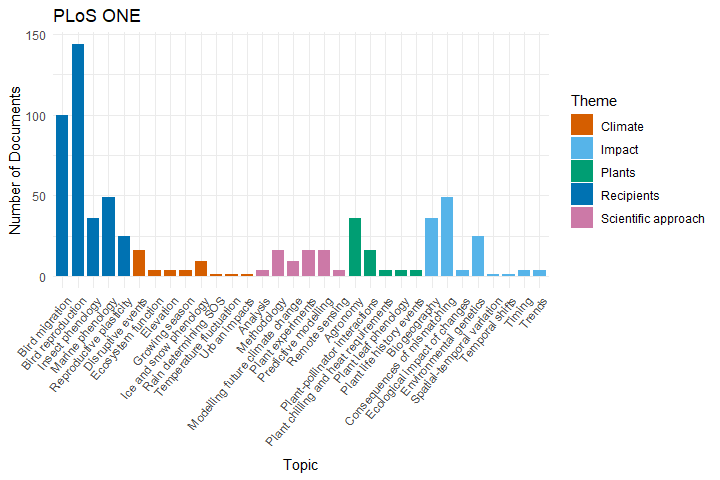


**Figure** **8:** The distribution of topics and themes among 117 documents for the journal PLoS ONE.


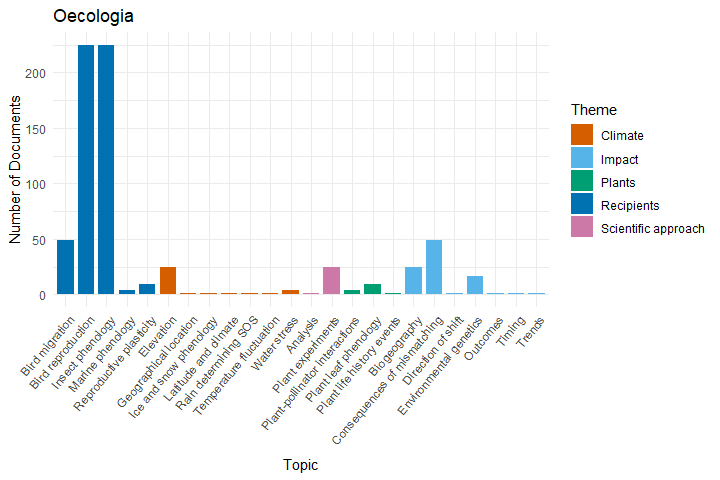


**Figure** **9:** The distribution of topics and themes among 86 documents for the journal Oecologia.

## Changes over time


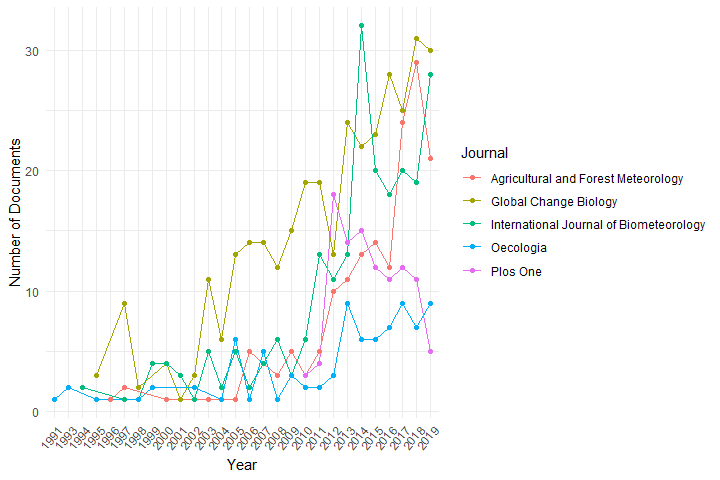


**Figure** **10:** The number of articles published in each year for each of the top 5 journals.


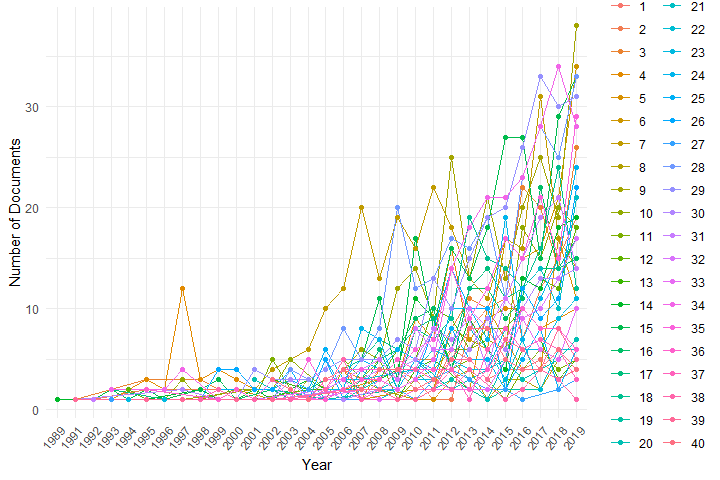


**Figure** **11:** The number of articles in each of the 40 topics over time.
